# Electroencephalography findings in patients with acute post coronary artery bypass graft encephalopathy

**Published:** 2014-10

**Authors:** Sadia Hanif, Shobhit Sinha, Khurram A. Siddiqui

**Affiliations:** *From the Department of Neurology (Hanif), National Neuroscience Institute, King Fahad Medical City, Riyadh, Kingdom of Saudi Arabia, the Department of Neurology (Siddiqui), Medical Institute, Al-Ain Hospital, Al-Ain, and the Section of Neurology (Sinha), Department of Medicine, Mafraq Hospital, Abu Dhabi, United Arab Emirates*

## Abstract

**Objectives:**

To determine the EEG findings associated with acute post coronary artery bypass graft encephalopathy (aPCE), and to study the demographics and neuroimaging findings.

**Methods:**

We reviewed the EEG in all patients with the diagnosis of PCE between February 2006 and December 2011.

**Results:**

We identified 21 (20 males, and one female) patients with aPCE. The mean age (±SD) was 64 (±11.2) years. Thirteen patients had altered level of consciousness, and 8 presented with confusion out of which 3 had acute seizures. The EEG patterns observed were: a) generalized theta plus intermixed diffuse delta in 7 (33%); b) generalized theta with focal epileptiform discharges in 5 (24%); c) generalized triphasic pattern in 3 (14%); d) generalized theta with lateralized delta in 3 (14%); e) generalized theta with periodic lateralized epileptiform discharges (PLEDs), and bilateral synchronous periodic epileptiform discharges (BIPLEDs) in 2 (10%); and f) one patient (5%) with electrographic seizures. On EEG/neuroimaging correlation, the EEGs that showed generalized slowing and generalized triphasic patterns had no acute changes on imaging, while the EEGs that showed lateralized slowing, focal epileptiform discharges, electrographic seizures and PLEDs had fresh infarcts. Patients with BIPLEDs had unremarkable imaging.

**Conclusion:**

The EEG features such as lateralized slowing, PLEDs, and electrographic seizure were associated with acute cerebral insults. An altered level of consciousness was the most common symptomatology in our cohort, and could possibly be related to hypoxic/toxic-metabolic etiology. Electrographic seizure detected by EEG may clinically present as aPCE.

Acute post coronary artery bypass graft encephalopathy (aPCE) varies widely from 8-32% of patients,^1^ and can be a major factor for prolonging hospital stay.^2^ Common factors predicting aPCE can vary from old age, history of hypertension, diabetes mellitus, carotid artery disease, and metabolic derangements.^3^ The aPCE is usually associated with short term and long term neurocognitive complications,^3^ so early recognition and appropriate treatment can help in better management of these patients. Electroencephalography (EEG) can ascertain a degree of encephalopathy, and also identify regional and global dysfunction that may help to point toward an underlying etiology. The EEG is most commonly used in patients with encephalopathies. Digital/computer EEG’s are perspective tools for mild ischemic brain changes in the postoperative period appearing as cognitive dysfunction.^4^ The aim of this study was to determine EEG findings in delirious patients post coronary artery bypass graft (CABG) in an acute setting.

## Methods

We carried out a retrospective observational analysis of patients who developed encephalopathy post CABG. We reviewed the EEG database at the Department of Neurophysiology, National Neurosciences Institute, King Fahad Medical City, Riyadh, Saudi Arabia from February 2006 to December 2011. Our study included all EEG’s carried out post CABG in patients who were admitted to the cardiothoracic intensive care units (ICU’s), coronary care units (CCU’s) and general ICU’s. We recorded demographics, day of diagnosis, symptoms of aPCE, encephalopathic features as per DSM-IV criteria (American Psychiatric Association) along with EEG and neuroimaging findings. The DSM-IV criteria for encephalopathy include: 1. Confusion/agitation; 2. Combativeness; 3. Alterations and fluctuations in levels of consciousness; 4. Acute problems in cognition including memory; and 5. Changes in perception including hallucinations.

This work was performed with informed consent of the subjects. Institutional Review Board approval was obtained for conducting and publishing this work.

## Results

**Figure 1:**
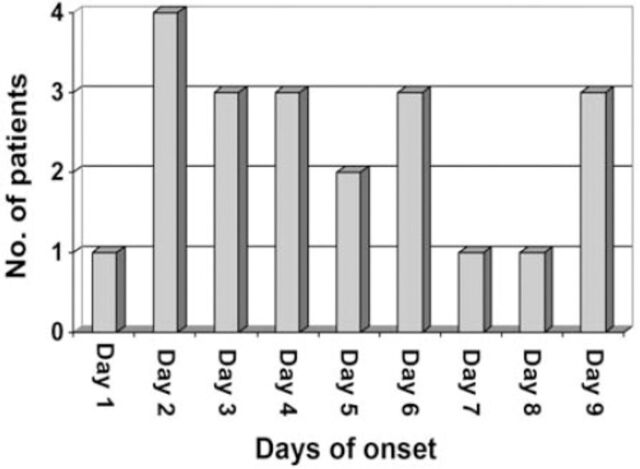
Days of onset of encephalopathic features with number of patients among post coronary artery bypass graft encephalopathy patients.

**Figure 2:**
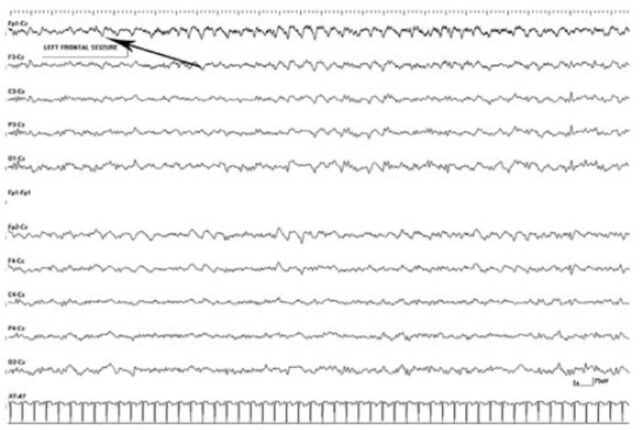
The EEG epoch showing left frontal electrographic seizure (arrow) in a 53-year-old post coronary artery bypass graft encephalopathy patient with acute left frontal infarct.

We identified 21 (20 males and one female) patients with aPCE. The mean age (±SD) was 64 (±11.2) years. The aPCE was diagnosed within a mean of 4.7 (range 1-9) days post CABG (**Figure 1**). Thirteen patients had an altered level of consciousness, and 8 presented with confusion out of which 3 had acute seizures. The EEG patterns observed were: (a) Generalized theta plus intermixed diffuse delta in 7 (33%); (b) Generalized theta with focal epileptic discharges in 5 (24%); (c) Generalized triphasic pattern in 3 (14%); (d) Generalized theta along with lateralized delta in 3 (14%); (e). Generalized theta with periodic lateralized epileptiform discharges (PLEDs), and bilateral synchronous periodic epileptiform discharges (BIPLEDs) in 2 (10%); and (f); One patient (5%) with electrographic seizure. On EEG/neuroimaging correlation, EEG’s that showed generalized slowing and generalized triphasic patterns had no acute changes on imaging, while EEG’s showing lateralized slowing, focal epileptiform discharges, electrographic seizures, and PLEDs had fresh infarcts. Patients with BIPLEDs had unremarkable imaging (**Figure 2**).

## Discussion

Our study showed that patients with aPCE who’s EEGs showed lateralized slowing, focal epileptiform discharges, PLEDs, and electrographic seizures had acute cerebral ischemic insults. Altered level of consciousness was the most common symptomatology in our cohort, and could be due to hypoxic, toxic, or metabolic etiology. Generalized theta intermixed with diffuse delta were the most common EEG findings in our study. Neurological assessment of the delirious and unresponsive patient is limited, only a few biochemical tests can provides clues, with significant limitations. However, electrophysiologic investigations provide a window into cerebral function, and are sensitive, quite safe, readily available, and inexpensive.^5^ Electroencephalography provides data that helps clinicians in decision-making in the setting of epilepsy related situations like hypoxic encephalopathy.^5^ Periodic lateralized epileptiform discharges on EEG are most commonly associated with cerebral infarctions, but may also be seen in encephalitis, tumor, or demyelination. Studies using computer EEG monitoring show that brain electrical activity recording demonstrates moderate sensitivity and high specificity in detecting brain ischemic alterations.^4^ A decrease in EEG amplitude, and or EEG slowing indicates a fall in mean cerebral flow below 22ml/100gm/min. An initial decrease in fast rhythms, namely, alpha and beta are followed by appearance of delta rhythms, and a further decrease in perfusion causes flattening of the EEG activity.^4^ Apart from the diagnosis of encephalopathy, the degree of severity can also be ascertained by EEG.

The degree of diffuse slowing of the normal background plus abnormal mixed rhythms in the EEG correlates with the severity of encephalopathy. Various studies have shown that EEG and neuropsychological deficits were found in almost 40% of patients after a week of CABG. It is estimated that worldwide the number of CABG’s carried out per year is around one million,^6^ and this number is increasing every year. The pathophysiology of post CABG encephalopathy is not very well understood, because there are many possible causes: infection, cardiac, or renal failure, and hypnotic medications. Diffusion weighted MRI studies indicate multiple small vascular lesions in patients after CABG, some of whom have reported to have encephalopathic features. The relatively old age of the patients who undergo CABG, and their widespread atherosclerotic disease are possible reasons for vascular sequelae particularly those leading to neurological dysfunctions.^3^ In our cohort, most of the patients had comorbid medical conditions increasing their risk of postoperative neurocognitive impairment. Post operatively, patients are intubated in intensive care units, which is a difficult setting for patients who develop aPCE and would require neuroimaging. We found that EEG is a simple tool that can be easily performed using portable EEG machines without delay.

### Study limitations

Our study was based purely on findings of EEG, which can point toward an underlying cerebral dysfunction, either global or regional. These findings have to be confirmed by neuroimaging, which reduces its specificity. In our study we did not look at whether patients who develop aPCE had a longer stay in the hospital than those patients who develop acute cerebral insults and seizures, and whether appropriate management dissipated the features of encephalopathy. Further study is required to address these points.

In conclusion, EEG features such as lateralized slowing, PLEDs, and electrographic seizure were associated with acute cerebral insults. An altered level of consciousness was the most common symptomatology in our cohort, and could possibly be related to hypoxic/toxic-metabolic etiology. Electrographic seizure detected by EEG may clinically present as aPCE.
